# Drivers of decision making in pain diagnosis and treatment: Findings from an ethnographic study of veterinary practice

**DOI:** 10.1111/evj.14562

**Published:** 2025-07-27

**Authors:** Rebecca Smith, Elizabeth Perkins, Gina Pinchbeck, Joanne Ireland

**Affiliations:** ^1^ Department of Equine Clinical Science Institute of Infection, Veterinary and Ecological Sciences, Faculty of Health & Life Sciences, University of Liverpool Liverpool UK; ^2^ Department of Primary Care and Mental Health Institute of Population Health, Faculty of Health & Life Sciences, University of Liverpool Liverpool UK; ^3^ Department of Livestock and One Health Institute of Infection, Veterinary and Ecological Sciences, Faculty of Health & Life Sciences, University of Liverpool Liverpool UK

**Keywords:** grounded theory, horse, horse‐human relationship, qualitative, sociology, welfare

## Abstract

**Background:**

Poor pain management in horses is a welfare concern. The ‘diagnosis’ of pain cannot be separated from the broader set of interactions through which it emerges. The interactions that take place during veterinary consultations shape the ways in which, or whether, pain management is discussed.

**Objectives:**

To understand owners' and veterinarians' decision making in relation to chronic pain and its management.

**Study Design:**

Qualitative ethnographic study.

**Methods:**

Data were collected from four veterinary practices in Great Britain between May 2023 and April 2024. Around 200 h of ethnographic observation was undertaken, including the observation of 47 consultations. Additionally, semi‐structured interviews with 18 veterinarians and 25 owners/carers were conducted. Principles of grounded theory were drawn upon throughout the collection and analysis of data.

**Results:**

This study focusses on the veterinary consultation, discussing how the interplay of actors—both human and nonhuman—affected pain diagnosis and treatment decision making. There were four key processes that were common across the consultations observed: ‘Establishing the presence of painful issues’, ‘Searching for the cause’, ‘The (de)escalation of issues of concern’ and ‘Targeting the source of pain’. In each consultation, dynamic multi‐actor interactions coproduced matters of concern, and these were the ‘things’ to which peoples' perceptions of pain were related. Pain diagnosis was not a ‘matter of fact’, but emerged through relational practices. Consultations could move towards a focus on the horse's management, however, other matters of concern could obfuscate discussions about the subjective experience of the horse.

**Main Limitations:**

Focus on leisure horse population.

**Conclusions:**

Pain could become a matter of veterinary concern if it emerged through unique contexts and practices. The nature of presentation to the veterinarian as well as the (re)construction of issues affecting the horse through veterinary medical practices could create tensions for pain management. Opportunities to improve practice are highlighted.

## 1 INTRODUCTION

Domesticated nonhuman animals (hereafter, animals) are reliant on humans to meet their welfare needs. Pain has been described as a multidimensional sensory and emotional experience, and alongside other factors can greatly affect an animal's welfare state.^1^ Early recognition and treatment of pain, as well as the management of its cause, are important for pain control. However, there are concerns about a lack of recognition of pain behaviours.^2^ In instances where a horse is presented to a veterinarian, it is unclear how diagnosis and treatment decision making is currently approached. Despite the potential significance of decisions made during veterinary consultations for pain management, there has been little sociological study of these encounters.

Veterinarians' work is performed within a paradigm that is focussed on diagnosis and treatment.^3^, ^4^, ^5^ The Veterinary Surgeons Act (1966) considers the diagnosis of disease as part of the ‘art and science of veterinary surgery and medicine’.^6^ The Royal College of Veterinary Surgeons (RCVS) Code of Professional Conduct requires veterinarians to promote animal health and welfare, and to consider the needs and circumstances of the client when formulating treatment plans.^7^ The RCVS state that, once a provisional diagnosis has been reached, treatment options should be communicated to the client. This framing by the RCVS suggests that appropriate treatment options are contingent on animal and owner factors, and that these factors hinge upon a diagnosis defined through the application of veterinary expertise. Whilst the construction of a diagnosis is framed as a ‘matter of fact’, from a sociological perspective, these processes cannot be separated from the broader set of interactions through which they emerge.^8^, ^9^ Diagnosis is inherently related to concepts of ‘health’ and ‘disease’ and these in turn shape ideas about the interests of the animal ‘patient’.^10^ Increasingly, the role of nonhuman actors, such as animals and technologies (to name a few), are also recognised as shaping interactions and social processes and therefore may be significant for how pain diagnosis emerges.^11^ While guidance around veterinary treatment decision making requires the consideration of a number of factors, there has been little empirical study of peoples' every day experiences, especially in the context of chronic pain management. Therefore, this sociological perspective offers a way to investigate how multispecies interactions affect the management of pain in the veterinary setting.

In humans, pain is a common driver for healthcare seeking, however, in the context of animals, other issues or behaviours which may or may not be related to pain may be viewed by animal owners as requiring the expertise of professionals. In a study of veterinarians treating horses diagnosed with primary back pain, owners often presented their horse for behaviour or performance related issues.^12^ An international survey of sports and leisure horse owners indicated that timing of veterinary involvement for lameness varies, with around half of respondents reporting they would manage the issue via other means before involving a veterinarian.^13^ Therefore, instances of animal pain identified within the veterinary consultation may be hidden behind other concerns and expectations of the animal's owner. In Belshaw et al.'s study of dog owners prior to the diagnosis of osteoarthritis, owners recalled one of two behaviour changes in their dogs that signalled there might be a problem.^14^ Belshaw states that ‘A few dogs showed acute changes, typically non‐weight bearing lameness on a single limb during or immediately after exercise, and more rarely vocalisation when moving or repeated licking of a joint or foot. This was always ascribed by their owners to the dog being in pain’.^14^ Other owners reported chronic, subtle, intermittent changes in gait and/or changes in their dog's demeanour. In the case of these subtler changes, pain was rarely described as a suspected cause. Presentation to the veterinarian resulted from owner observations but prior to this could involve a period of ‘wait and see’ and/or ‘lay treatment’ and/or ‘informal help seeking’.^14^

Whilst inevitably shaped by their veterinary training, veterinarians' attitudes and approaches towards pain evaluation and management in horses varies.^15^, ^16^, ^17^ Previous survey‐based studies have used vignettes of clinical conditions or surgical procedures to investigate veterinarians' approaches.^15^, ^16^, ^17^ However, these hypothetical scenarios do not allow for exploration of the ways in which decision making actually emerges in practice. Furthermore, taken for granted assumptions held by participants, or contextual factors meaning that intentions may differ from behaviours, cannot be captured. While pain may arise in many contexts, painful musculoskeletal conditions such as osteoarthritis, chronic laminitis and back disorders are prevalent across the equine population and welfare concerns may arise in relation to how these issues are managed.^18^, ^19^, ^20^ In this study, the concept of chronic orthopaedic pain was adopted as a lens for exploring pain diagnosis and treatment in the veterinary setting.

The wider study on which this study is based sought to understand owners' and veterinarians' decision making in relation to chronic pain and its management. The study adopted an ethnographic approach to investigate how and why pain becomes a matter of concern in veterinary consultations. In doing so, the study aimed to produce theoretical insights that explain how decisions affecting chronic pain management are shaped. This paper focusses on the veterinary encounter, whilst owners' conceptualisations of chronic pain and veterinary advice‐seeking behaviours are presented elsewhere.

## 2 METHODS

This study adopted an ethnographic methodological approach. Ethnography involves observing the behaviour of participants in a given social situation aiming to generate rich insights into people's understandings and actions. The study adopted a symbolic interactionist theoretical perspective as its starting point. In doing so, the study drew upon the premises that meaning arises through interactions and that people act toward things based on perceived meaning.^21^ This perspective has been used to study the ways in which human‐animal relationships generate understandings of animals, including the ways in which animal subjectivity or emotion is given meaning.^22^ Ethnographic methods of data collection use observation to record in detail social interaction and the perspectives of participants, and to understand these in their local contexts. It has been described as ‘the study of people in naturally occurring settings or “fields” by methods of data collection which capture their social meanings and ordinary activities’.^23^ Considering the ways in which contexts and practices shaped interactions offered a way of understanding how attitudes and approaches towards pain diagnosis and treatment emerged.

The use of constructivist grounded theory methods of data collection and analysis in this ethnographic study was aligned with the symbolic interactionist theoretical perspective. This approach assumes multiple realities and emphasises how meaning and action are co‐constructed, including knowledge creation between researcher and participant.^24^ The presence of the researcher—Rebecca Smith, hereafter RS—alongside participants in the field enabled in‐depth understanding of experiences, as well as observation of context and behaviour. Throughout the study, reflexivity was important and ongoing. This involved reflection on RS's own beliefs and assumptions and role in the research process, and is discussed further below.^25^, ^26^ This study sought to obtain an in‐depth, comprehensive, and coherent understanding of a specific area. From the constructivist perspective, the results presented are understood to be an interpretation, or reconstruction, of studied life.^25^

### 2.1 Data collection

Veterinary practices in Great Britain that provided ambulatory services for predominantly leisure (non‐professional) horse owners were recruited. Purposive sampling of practices was undertaken to include a variety of geographical locations, practice size, solely equine and mixed companion/production animal practices, access to hospital facilities, and privately or corporate owned. Recruitment involved contacting practices by email, phone call, or visit, and some via gatekeepers known to the research team. Potential practices were provided with a participant information sheet outlining the study's aims and methods, with the opportunity for discussion with RS. In total, four practices were observed, each for a period of 1–2 weeks. Length of observations was partly dependent upon practice availability and factors including the scheduling of owner interviews. Ethnographic work was undertaken by RS, a female veterinarian who worked previously in small companion animal practice with experience of caring for (but not owning) horses. RS had completed a PhD and was trained in social research methods. RS had previously met two of the participating veterinarians during the course of her work, but in other cases, there was no prior relationship. The recruitment of practices continued alongside early data collection. Data were collected between May 2023 and April 2024. This was to ensure that experiences were captured across the different seasons, as weather and seasonal differences in horse keeping practices were potential factors of significance. All staff at the veterinary practices were informed about the study and its aims. Participants gave their informed consent to participate in the study.

Within each practice, consultations to be observed were selected in discussion with veterinarians to capture relevant data on the basis of the stated purpose, for example, lameness, stiffness, vaccination of older horse, euthanasia. These types of visits had been identified as likely to be relevant to the study based upon an initial literature review and the authors' experience of veterinary work. In a couple of instances, the veterinarian declined for RS to attend due to the sensitivity of a consultation, for example, euthanasia. As analysis developed, consultations were also selected using a theoretical sampling approach in order to explore variation within developing categories (see data analysis). In some instances, additional veterinary staff were interviewed without observation of a specific consultation to enable exploration of past experiences relevant to the topic of study and to capture ranging demographics, for example, time since qualified. Horse owners were recruited for the observational component upon introduction by the veterinarian. During the initial consent‐taking process, where the topic of the study was introduced, requests for a follow‐up interview were made. Owners were generally happy for the consultation to be observed, and all but one agreed to be contacted at a later time for an interview.

Field notes were compiled following informal conversations with veterinary staff, veterinarians, and owners, and after consultations and recorded interviews. This entailed documenting brief notes on a phone or writing these up more fully on a laptop once back at the practice or later that evening. Notes contained observations of conversations, examinations, and impressions of the site. In a separate column, impressions and early interpretations were also documented.^23^ Initial field notes were extensive, and everything that may have been important was noted, and over time, they became more targeted.

Ethnographic interviews with veterinarians (conversations with participants in the course of their work) took place in the car between visits; some of these interviews were recorded. In some instances, additional interviews were undertaken when back at the practice, and others were arranged for a later date, for example, in the weeks following, and one was delayed by several months due to veterinarian availability. Whilst RS sought to contact participating owners in the day(s) following the consultation to arrange the interview, these were often conducted the following week. The timeframe between the consultation and the interview ranged from immediately following the consultation to 5 weeks in one case. Some owners who agreed to be contacted for interviews did not subsequently participate, and in some instances they were not contacted due to the sampling approach or otherwise failed to participate due to lack of response. Owner interviews were held in person at the horse housing premises, for example, livery yard or owner's home, on‐site at the veterinary practice if the owner attended with their horse, or by phone call or video‐based platform. In one instance, the owner preferred to answer questions about her experiences via email, which enabled reasons for booking the veterinary visit to be explored; however, it did not gather the same degree of detail as interviews.

Interviews followed a semi‐structured approach using owner/veterinarian topic guides (Text S1). Conversations centred on the horse(s) attended to during the observed consultation. However, for owners, additional current or previously owned horses were sometimes discussed, and for veterinarians, past experiences and approaches to pain management in additional cases were also explored. Questions varied depending on the individual's experiences, and as analysis developed, questions were adjusted. The interview duration ranged from approximately 20 to over 90 min. Participants were provided with an information sheet and contact details for the research team. Interviews were audio recorded and subsequently transcribed by a commercial transcription service that uses human transcribers and an intelligent verbatim approach. Transcripts were anonymised and pseudonyms used throughout.

### 2.2 Data analysis

Data analysis took place alongside its collection, drawing on the principles of grounded theory.^25^ Data analysis was primarily undertaken by RS, with review of data and emerging analysis also performed by Elizabeth Perkins (EP), Gina Pinchbeck (GP) and Joanne Ireland (JI). An inductive approach to coding meant that salient categories and concepts were developed from participants' accounts and researcher observations. Diagrams representing categories and relationships were used throughout analysis and were documented on paper and software (NVivo 12). Data collection and analysis were ceased at a point where a detailed account of the data was achieved.^27^ The inclusion of a range of consultation types enabled variation to be explored. The constant comparison of data enabled similarities, differences, and relationships to be explored. Early data analysis enabled theoretical sampling in order to test and refine categories. For example, it was hypothesised that an owner's desire to access medication under veterinary control drove advice‐seeking behaviours; therefore, a consultation for a repeat prescription of a non‐steroidal anti‐inflammatory drug (NSAID) for a horse with osteoarthritis was selected for observation and the owner recruited for interview. This approach enabled the development of theoretical statements that connect categories and explain patterns.^23^

### 2.3 Reflexivity

As RS navigated relationships with participants, rapport building often involved disclosure of elements of her past history as a veterinarian and interest in the topic of study. Whilst being aware that her veterinary background facilitated relationship building, the role as observer rather than provider of veterinary expertise was something that had to be managed throughout interactions with participants. Furthermore, the effect of her scientific training on the interpretation of data was reflected upon by RS. Understandings of scientific concepts and ideas about where questions concerning pain might arise in veterinarians' everyday work did not fit with the data collected in the field. In addition, the implicitness of the meaning of pain across the data was at first confusing. However, discussion with LP, GP, and JI at an early stage enabled further questioning and reflection. This highlighted how experiences and processes were shaping interactions, and at the same time this meant that certain aspects of concern—such as pain—were muted or not explicitly discussed.

## 3 RESULTS

In total, around 200 h of overt non‐participant observation was undertaken, including 47 veterinary consultations. In addition to semi‐structured interviews with 18 veterinarians and 25 horse owners or carers, conversations with a further 12 veterinarians, as well as reception staff and veterinary nurses within the practices, were completed. Veterinary practice details are detailed in Table S1 and owner interview participant details are detailed in Table S2.

Four key processes emerged throughout veterinary consultations and these shaped diagnosis and treatment decision making. These will be presented as follows: ‘Establishing the presence of painful issues’, ‘Searching for the cause’, ‘The (de)escalation of issues of concern’ and ‘Targeting the source of pain’. Whilst these processes were common across the consultations, individualised sets of multi‐actor interactions meant that the outcomes of interactions varied. The nature of pain diagnosis and treatment was not a ‘matter of fact’, but emerged through and was contingent upon, these dynamic interactions.

### 3.1 Establishing the presence of painful issues

The description of the consultation in the practice booking system informed veterinarians' ideas about the nature of the owner's concerns as well as what their veterinary approach might require during the consultation. There were two overarching—and potentially overlapping—categories of consultation type: (i) ‘routine’ health care such as dentistry, vaccination, sedation or a check‐up associated with long‐term prescription medication (e.g., NSAID, pergolide), or (ii) consultations for an ‘issue’ related to the horse's health. Some were defined as the initial presentation to a veterinarian, while others had been preceded by other consultation(s) for the issue by the same or other veterinarian(s). The reception teams' documentation of the issue of concern as well as any personal interactions a veterinarian had with the horse's owner, played a role in framing the veterinary visit. The extent to which this information was documented in veterinary records varied. There was also variation in the extent to which the initial framing of the horse's issue aligned with subsequent veterinary diagnosis:

I mean, lameness is such a broad church. It really does cover everything from a hoof abscess to a broken leg and anything in between. (Veterinarian, Practice C)

Pain per se was generally not the initial reason given by the owner as the need for a veterinary visit. However, through experience, veterinarians came to recognise patterns in how, or when, animals in pain might be presented to them. While chronic pain was not in itself considered to be a trigger for a consultation request, owner‐reported concerns about changes in behaviour or movement were said to alert veterinarians to underlying pain:

The presenting words or phrases that a client would use, that would alert my brain, and make me think, ‘Maybe this is a painful animal’, would be things like changing behaviour, poor performance, or lameness. My understanding of lameness is pain. But that's not the case, I think, for every client. (Veterinarian, Practice D)

Over the course of the year, seasonal fluctuations and associated medical conditions could also result in animals with recurring pain being presented to veterinarians:

laminitics that are, effectively, the same time every year, i.e. cases that get intermittently sore. (Veterinarian, Practice B)

While underlying pain was variably the trigger for veterinary advice‐seeking, it could nevertheless form part of an owner's concern about their horse:

So yeah, she arrived. And she was actually really angry. Really, really, really angry. We were wondering what was going on, if she was sore. Or, ‘Is this just her?’ We didn't really know…But we did ask for her to be, sort of, evaluated as, you know, what do the vets think? Does she look sound? Do they see anything? They couldn't really see anything obvious, but it was suggested that she might have ulcers. (Owner, Practice A)

Views on what constituted a painful issue could differ between owners and veterinarians. One veterinarian mentioned that certain requests for, or use of, pain relief by owners were deemed to be unjustified:

‘Oh, normally he's on one bute [NSAID], once a day, but the farrier came yesterday, so I gave him two’. And you think, ‘Well, he shouldn't need more. The farrier should not be painful. He shouldn't need pain relief when he sees the farrier. He shouldn't need pain relief when he sees the dentist. It's not a painful procedure. He shouldn't experience discomfort from it’. (Veterinarian, Practice D)

Through speaking to the person presenting the horse and observing or examining the animal, veterinarians negotiated the services required of them. The length and scope of conversations about the horse and their management was affected by time availability, individual conversation styles (sometimes open, but more often closed questions were observed being used), the extent to which owner and veterinarian knew one another, the veterinarian's position within the practice and their perceived role in the ‘case’:

I mean, still now, if I ever see a horse that's not my case, not my client, I'm just there for a recheck because the other vet can't come, for whatever reason, I always just try to do minimal communication, make sure everything's okay, and then I just report back to the case vet, and say, ‘Oh, maybe give them a call’, or something like that. (Veterinarian, Practice D)

Whilst veterinarians' approaches were key to shaping the exchange of information, owners' approaches also drove this process. To aid in the delivery of relevant information, one owner had listed their concerns on paper which was brought to the consultation and referred to during the history taking process. However, whether and to what extent an owner's concerns were discussed during consultations varied. During one observation, the owner's concern about pain was only raised in the latter part of the interaction:

Following an examination of the horse, the veterinarian talked about a degree of improvement since her last visit and that from her perspective she was happy to continue management, including the use of pain‐relieving medications, and re‐assess the horse at a later time. At this point, the owner's aunt asked ‘He's not in pain is he?’ and the owner made gestures of agreement with this concern. (Field notes, Practice B)

Veterinarians' frame of reference for pain assessment stemmed from their intermittent involvement with an animal—either through routine health care provision or issues raised by the horse's owner—and so assessments were based upon these medicalised contexts in which horses and their health were observed. Establishing the presence of a painful issue was a process that emerged through varying degrees of physical examination. In practice, examinations ranged from observations alone to more extensive palpation of a horse's limbs or back, percussion of hooves, observations of the horse moving or being ridden, or anaesthesia of nerves and/or joint(s). Whether and in what way these types of examinations came about affected veterinary identification of matters of concern.

Crucially, examinations played a role in both establishing the presence of and searching for the cause of a painful issue. However, the extent of examinations was contingent upon a number of context‐specific factors. In interviews, some veterinarians talked about getting an ‘initial impression’ of a horse and their condition based on interpretations of their behaviour, for example, facial expressions, posture, demeanour or movement, and this was said to play a role in guiding further examinations. The necessity of performing particular diagnostic procedures was primarily influenced by the veterinarian's knowledge and beliefs about their potential usefulness, in conjunction with any owner requests. For example, during a repeat prescription check‐up for a horse with navicular disease, it was the suggestion of the person presenting the animal (the owner's friend) that the veterinarian observe the horse being walked out that precipitated the assessment.

Veterinarians' examinations relied upon the differentiation of ‘normal’ from ‘abnormal’ and a baseline knowledge of the horse was reported to assist in this. Some also discussed the need to navigate context‐specific factors when making inferences about whether an ‘issue’ existed. These factors included the potential effect of ‘mechanical‐based lameness or gait asymmetry’ (Veterinarian, Practice B), or reproductive cycles in mares. There was thus complexity in terms of identifying issues, but also in ascribing any association this might have with underlying pain. Some veterinarians spoke about the value of an owner's knowledge of their horse in assisting with this, and a few involved the owner in observing and commenting on the horse's behaviour or movement during examinations. However, when making inferences about the horse's subjective experience, including whether they were in pain, veterinarians generated expectations in line with the presence of medical conditions:

I think we have an idea of how painful certain conditions are, I guess. So, what you're expected response and reaction to that condition might be, although that can be very individual. (Veterinarian, Practice D)

During interviews, veterinarians often considered the presence or absence of pain to be a ‘matter of fact’. However, when asked directly about what they thought chronic pain was, a number found it challenging to answer. Rather, when describing what pain might mean for a horse, veterinarians drew upon medicalised understandings of the body and disease, their own personal experiences of pain or owner concern. One veterinarian spoke about horses and ponies displaying pain differently, and that potentially there was variation across breeds. Pain was talked about in relation to its duration and intensity, for example, background pain or flare‐ups, and in a few instances, about pain being potentially persistent or something that may resolve. While veterinarians reported that owners often experienced difficulty in identifying pain in their horse, some also questioned whether horses learned to live with or tolerate pain over time. There were some difficulties in articulating how chronic pain might be differentiated from more acute episodes. There was no consensus about how long pain should persist until it is classed as ‘chronic’: a number of veterinarians considered that it was the inability to resolve pain meaning it continued over a number of weeks into months.

In some cases—generally of acute or surgical‐related illness involving hospitalised patients—attempts to formalise pain assessment involved the use of pain scoring frameworks. It was considered by senior veterinarians introducing these tools within their practice that these acted to raise pain as a particular focus of concern during veterinary examinations. Nevertheless, time constraints were reported to be a barrier to uptake even within hospital settings, and there was no evidence of their use in ambulatory practice.

The identification of issues, rather than pain per se, drove decision making in veterinary diagnosis. Not all consultations led to pain becoming a matter of concern, and this was the case even where the presence of a potentially painful condition had been raised. In the sections that follow, the search for the cause of the horse's issue and (de)escalation of issues of concern are discussed. These processes had implications for whether, and in what way, consultations came to involve pain management discussions.

### 3.2 Searching for the cause

The framework underpinning veterinary work encompassed identifying and treating painful problems, and this meant searching for their cause:

But, obviously, you know, when a horse goes lame, normally it is a sign that there is pain somewhere. We need to locate it, we need to numb it, and then we are going to know where the pain is coming from. (Veterinarian, Practice D)

This search was also driven by owners, especially where veterinary advice‐seeking was preceded by previous (unsuccessful) attempts to remedy issues with a horse's health or behaviour. This meant that owners had certain expectations of veterinary service provision:

Someone who is able to get to the crux of the matter quickly. And I know that's not easy. But to be able to identify what's wrong and respond to it. (Owner, Practice D)

The degree to which investigations were carried out varied depending on these context‐specific factors. These included ideas about the suspected cause, the horse's age, breed, owner's wishes, financial resources or competition goals, and the practicality of access to onsite or referral diagnostic facilities.

Extensive diagnostic ‘work‐ups’ were not always undertaken, or deemed necessary. These could become more targeted in instances where a horse presented for a follow‐up examination where a diagnosis had already been defined. The practices visited had access to facilities including arenas and areas for trot‐ups (including soft and hard ground), as well as diagnostic imaging. In some cases where previous attempts to remedy the issue had not been entirely successful, horses could be admitted for the purposes of clinic‐based investigations. However, even where more extensive diagnostic procedures were carried out, questions about a medical cause could remain.

In the context of a study focussed on chronic pain, it became clear that ability to identify and localise the cause of a horse's movement issue was important for equine veterinarians. As veterinarians could build their reputation and business based on their proficiency in lameness investigations, establishing this expertise had professional significance. As discussed later in 3.3 The (de)escalation of issues of concern, differing views on the presence or meaning of movement abnormalities could also be a source of conflict between owners and veterinarians. The process of becoming a skilled equine veterinarian was exemplified during one lameness work‐up in a veterinary practice, when the senior veterinarian asked junior staff present, as well as RS, what we thought about a horse's behaviour and movement following regional anaesthesia of a joint. To what extent had the joint block returned the horse to ‘normal’, and could this knowledge guide action in remedying the issue? For veterinarians, the presence of pain was perceived to be linked to a problem that—with sufficient expertise and resources—could be identified and in some way managed.

The search for the cause of an issue was also shaped by nonhuman actors with technology, such as radiography or ultrasonography, acting in ways that drove a search for a diagnosis. Diagnostic imaging could make the body and its diseases ‘visible’. It could make visible the cause of pain and assist in being able to direct treatment of disease. In doing so, these interactions could (re)construct, or bring tension to, how the owner understood their horse and their health:

The images were instantly available and the veterinarian looked at these and discussed together with the owner his findings—significant mottling, spurs and bony remodelling across the joint. The owner asked whether that could account for the lameness seen, she was also unsure why the lameness didn't develop until the following day after trotting the day previously. (Field notes, Practice A)

Deciphering the degree to which underlying pain was related to the issue at hand, as well as its biological nature, could involve the use of pain‐relieving medication in the diagnostic pathway. As one veterinarian explained:

if you get a positive response, that's really beneficial, but if you get a negative response, and what I tell people at the very beginning is, ‘You get a negative response, it just doesn't necessarily mean that this isn't a painful condition. It just means the pain isn't responding to a non‐steroidal anti‐inflammatory’. And if you have neuropathic pain, if you've got another type of pain, then you just really have to factor that in. (Veterinarian, Practice D)

This scientific knowledge relating to the neurobiology of pain and pharmacological interventions acted in ways that drove both a search for, and also the treatment of, pain. However, during conversations with veterinarians, discussions about pain pathways and how chronic pain might differ from more acute episodes were fairly limited.

### 3.3 The (de)escalation of issues of concern

Where multiple issues were constructed during the veterinary encounter, they did not all become a focus of concern. The processes of diagnosis led to certain issues being (de)escalated as areas for exploration at that time. In one example, a horse presented with hindlimb lameness, and during the consultation, the owner queried whether the horse had arthritis. The veterinarian's subsequent detection of a foot abscess was said to explain the issue at hand. The veterinarian commented that arthritis was common in older horses and that it was not a major issue for this horse that would warrant long‐term analgesia at this time. As a result, review of the horse's mobility and comfort was left for a future visit:

The criteria for that, I suppose, one was finding a foot abscess, and treat what we know we have rather than what we might have. I think certainly when I flexed her hock you could feel a little bit of resentment. And when she walked away, she did look a little stiffer, and it might be one that's worth another look at, once this has sorted itself out. (Veterinarian, Practice C)

While the owner's narrative around the presenting issue played a pivotal role in the initial framing of the consultation, the veterinarian directed the way in which issues were prioritised and dealt with at the time. In another example, despite the owner raising concerns about her pony's comfort level and whether he should be receiving pain relief in older age, the veterinarian did not perform a full examination:

But that wasn't what we were there for. We were there to blood sample for the Cushing's, weren't we? Yeah, so it might have been one of those things where the client wasn't expecting that from us anyway, but it just might have been a nice, extra bonus that we could have done. (Veterinarian, Practice A)

The presence of other parties during the consultation impacted on the ‘issues’ constructed. In one case, the veterinarian and farrier attended a horse that had previously been diagnosed by the veterinarian with laminitis. In the car afterwards, the veterinarian described her interpretation of the radiographs as indicating significant changes indicative of some degree of chronic laminitis, and possible long‐term pain. However, she reported that she did not raise this point specifically due to concerns that it might have implied ‘poor’ farriery, and thus may have affected the veterinarian‐farrier relationship. Thus, relationships affected the way in which this diagnostic information was relayed to other parties involved in the horse's care. In theory, this may have implications for decision making concerning the animal's interests.

Nevertheless, veterinarians commonly perceived their role in consultations to involve raising concerns and/or communicating the magnitude of a horse's issue or suffering to an owner. Means of ‘escalating’ issues that were not obviously of concern to the owner included the use of analogies such as comparing to human experience or using direct language or warning of likely decline. There were examples of veterinarians trying to involve the owner in observations and reflections on the horse's behaviour or movement in attempts to elicit concern and action. However, broaching issues of pain was also acknowledged to be potentially emotive. The perceived severity of the issue played a role in how veterinarians balanced raising the issue to a concern whilst keeping the owner engaged with their services:

There's making somebody feel guilty about their inability to recognise pain. It's not going to get anybody any further forward in trying to help the horse. (Veterinarian, Practice D)

The (de)escalation of issues of concern not only had implications for the horse's access to investigation or treatment at the time, but longer‐term implications in relation to the veterinarian‐owner relationship. Differing views on the presence of an issue or its significance for the horse's wellbeing could affect owners' views of veterinary expertise and their subsequent advice‐seeking behaviours. One owner reported that her veterinarians' judgement of her horse's movement as being ‘just field lameness’ during a previous visit had not reflected her understanding of the issue, or concern about her horse:

he was lame and he kept putting his leg out. He wouldn't put weight on it. He wasn't bright. I'm not overly impressed with that vet, I'm afraid, because they turned around and said he had field lameness. I said, ‘Well what's that?’ They went, ‘Oh, it's just field lameness’. I said, ‘A horse is not… Field lameness means nothing’. They would say, ‘Well, you know, he's old’. They wouldn't give‐ They've got a problem with giving you a definitive answer, which is quite frustrating. (Owner, Practice D)

In this case, the owner sought advice from an alternative veterinarian whom she felt had particular expertise in this area. This example highlights the importance of achieving a shared understanding between the veterinarian and the owner about the horse's wellbeing and this involves clear and open communication. A decision to escalate or de‐prioritise an issue requires the veterinarian to communicate with an owner if they are to avoid owner dissatisfaction with the consultation.

### 3.4 Targeting the source of pain

Consultations *could* move towards a focus on treating or managing the source of pain. Understandings of disease and its anticipated trajectory framed veterinarians' ideas about how chronic pain might arise, and how it might be managed:

I feel that with chronic pain you are talking more about maybe degenerative diseases, that can't be solved, if that makes sense. It can maybe be slowed down, or prevented, but once they've started, there is no stopping it. (Veterinarian, Practice D)

Approaches to targeting the source of pain could span medical, surgical or husbandry‐related interventions. The veterinary diagnosis, perceptions of the owner's goals for the horse's lifestyle, finances, and horse age shaped the nature of veterinary advice:

I think the only thing, I guess, that I would be trying my best, in a young horse in particular, to try and stop the pain from becoming chronic. So, I think in a young horse, my focus is to very definitely try to identify and either fix or manage that problem early on. But to try and stop secondaries sequelae of pain occurring. So, I don't think it would change what I would do, but I very much have that in the forefront of my mind, that, for me, is a real goal. (Veterinarian, Practice D)

Analysis suggests some complexity underlying decision making regarding the prescription of pain‐relieving measures. In some instances, pain relief was not deemed appropriate from a veterinary perspective. In animals with foot abscesses—an acutely painful condition—analgesia was variably prescribed with some veterinarians raising concerns about the potential for delaying disease resolution, whilst others talked about it being part of their standard approach. Treating disease—rather than necessarily achieving early control of pain—appeared to drive decision making.

Pain management formed one part of (often standardised) veterinary approaches to treating health conditions. Oral NSAIDs were most commonly prescribed in association with a range of underlying issues. Sometimes, veterinarians directed the owner to administer medication they already had or prescribed a course. In some instances, it was the owner's request for pain relief that prompted veterinary prescription:

Following an examination and discussion about further diagnostic investigations in a consultation for a teenage mare presented for lameness, the veterinarian advised monitoring and reassessment in 10 days upon which the owner enquired about giving the mare some bute. The veterinarian agreed and went to the car to dispense some analgesic medication. (Field notes, Practice A)

Deciding what to prescribe entailed veterinarians making judgements about the nature and severity of the horse's pain, whilst also considering whether future escalation might be required:

I have seen probably enough laminitics now to know when just bute on its own will do the job with a combination of other management factors, but in this case, I felt like even intravenous bute and oral bute, going forwards, wouldn't probably be enough, so I added in paracetamol. I kind of tend to keep in my arsenal, kind of thing, so I wouldn't always add it to start with because you like to have another line, basically, that you can add in so owners feel like they're doing more if it's needed and if the horse declines in themselves. But in this case, I felt like we needed it as a first‐line treatment. (Veterinarian, Practice B)

The management of pain was always associated with other matters, with varying priorities or concerns shaping management approaches. Context‐specific factors could mean that certain options were preferred by owners, and preferences were not necessarily related to cost:

Respondent Or is it actually, could we‐ I'd rather inject into a joint than have a daily bute, personally.

Interviewer Okay. And what would be your reasons for that?

Respondent Management and practicality of it…There's 25 horses to manage. And I just find I'd rather pay a little bit more, and know it's sort of like‐ and not have to worry about it, as it were. (Owner/carer, Practice D)

In some instances, the need for pain relief was agreed upon by both parties, and yet, queries about how to safely manage pain were raised by owners. For example, a few owners raised concerns during consultations, or in interviews, about the long‐term use of NSAIDs in horses and the animal's gut health. Veterinarians raised problems relating to a lack of analgesic options available for horses, ‘appropriate’ use of analgesics by owners, and concerns relating to ‘masking’ pain and disease progression in some cases. Therefore, decisions about how to *appropriately* manage pain were situated within a broader context that involved interpreting its current significance as well as how potential harms to the horse's health and welfare more broadly might be prevented.

In some circumstances, such as the presence of co‐morbidities or owner concern about the practicality of a management plan, veterinarians adapted advice from what might be considered ‘best’ from a physical health perspective. For example, treating a horse ‘as a whole’ could mean giving the horse access to space and opportunity for movement to ease joint‐related pain, despite risking potential disease progression. Adjustment of workload or activity type was considered (often by more experienced veterinarians) to be an important part of long‐term management and could assist in maintaining a horse's fitness, functionality and comfort, as well as associated pain. There were examples of veterinarians drawing on their personal experiences of pain when considering how to best manage a horse's pain in the long term:

So, yes, I do think he's in background pain, but I think an acceptable level of pain. If I can frame that. Saying that, as I say, I have chronic back pain which I live with. Some days it's better. Some days it's worse. And actually, it's terrible for me if I spend all day sitting at a desk, and it's better if I'm gently moving it. (Veterinarian, Practice B)

As the veterinarian above explains, her understanding and experience of the fluctuating nature of chronic pain has shaped her views about the acceptability of the horse living with pain. One newly graduated veterinarian talked about how his veterinary training had made him view animals in a different light from how he had done previously. Through life experience and greater understanding about people's experience of pain, veterinarians also spoke about developing a greater awareness or consideration about what pain might be like for an animal.

There was a degree of inevitability about the progression of chronic pain, and in some respects its management was seen as part of caring in the context of anticipated decline:

And I did say to her that, ‘Unfortunately, the drugs are good, but they're not that good. As the osteoarthritic change gets more severe it is likely that we will need to do these injections more frequently to keep him comfortable. And she is well aware that it will get to a certain period when maybe they're not doing a good enough job, and we might need to have a discussion at that point. She's very sensible in that regard’. (Veterinarian, Practice A)

Pain and suffering were associated with concerns about horse welfare and raised moral questions about the ‘right’ thing to do. Where euthanasia was constructed as a veterinary ‘treatment’ option, pain also created contexts of end‐of‐life decision making. During a consultation where the owner questioned whether her horse was in pain, the veterinarian's response alluded to her interpretation of the presence and significance of pain for the animal in light of the ‘treatment’ options available:

The veterinarian responded by saying that she said would tell them if she thought they should put the horse to sleep, but given the improvement seen since the last visit, the degree of ambiguity around the cause of the mobility and neurological issues and therefore unclear prognosis, that she thought it appropriate that management of the horse continue. (Field notes, Practice B)

Palliation of pain was deemed permissible in some cases in light of the horse's functional capacity, response to veterinary intervention, and the horse's management context including the type of activities (e.g., ridden) the horse was involved in. Whilst owners were generally tasked with monitoring the horse, specific details about how pain in that individual animal could be assessed or monitored were not explicitly discussed, even where an owner specifically raised a concern. There was often little specific discussion about how, or in what ways, to interpret or monitor the horse's response to treatment—and whether they were in pain.

Despite this, the onus was on owners to seek further veterinary advice if required, often with an acknowledgement that the owner knew the horse best. In some cases, veterinarians discussed a plan for veterinary follow‐up, either in person or by phone; in others, they advised the owner to get in touch if they had concerns or anything changed. Nevertheless, the nature and timing of advice‐seeking behaviours, whether or not owners paid their bills and followed veterinary recommendations, were all judged by veterinarians and shaped the veterinarian‐owner relationship. The way in which owners raised issues of veterinary concern shaped whether, and in what way, veterinarians could carry out their work.

## 4 DISCUSSION

During four key processes in the veterinary consultation the interplay of actors affected pain diagnosis and treatment in various ways. Concerns about pain were always associated with, and distributed throughout, other matters. It was the construction of these other matters that affected whether pain became a ‘thing’ of concern, or not. Whilst understandings of the relationship between the horse's ‘issue’ and pain shaped decision making, interpretations were generally not made explicit. Veterinarians' attempts to move the consultation towards treatment or management conversations was affected by these interpretations as well as a whole host of contextual factors. The methodological approach adopted in this study brings in new ways of thinking about how animals, and their experiences, come to be known in the veterinary encounter. In doing so, it brings to light the ways in which veterinary consultations create and shape understandings of animals, as well as the decisions that affect their care.

The structure of veterinary consultations involved initial stages of information gathering and examination of the animal followed by varying provision of treatment or management advice. While this reflects the structure reported in small and farm practice, and that of the Calgary‐Cambridge model,^3^, ^28^ our study indicates that the construction of matters of concern was contingent upon factors beyond communication or examination practices alone. We identified that relationships between owner, veterinarian, and horse,^29^ biomedical representations of disease,^30^ and technologies,^31^ all interplayed in interactions. Furthermore, there was evidence that owners and veterinarians not only constructed the horse and issues of health and disease in differing ways, but that they also affected one another and coproduced painful problems.^30^ In these interactions of multiple actors, issues became entwined and could be raised or deprioritised as issues of concern. These matters of concern were the ‘things’ to which understandings of pain were always related, and as such their construction had implications for pain diagnosis and treatment. This illuminates the complexity surrounding chronic pain management suggesting that instead of being reliant on its ‘recognition,’ diagnosis and treatment practices are context‐specific and subject to a great deal of complexity. Where clinical decision‐making and the determination of what is in the animal's best interests rests upon this complexity, it matters not only clinically, but ethically.^10^

Findings suggest that in practice, there is no clear delineation at which pain becomes ‘chronic’. In the course of their work, veterinarians were presented with horses with painful issues that could be, or could become, a long‐term concern. Pain was perceived as a clinical sign secondary to something else, and was talked about in relation to (mal)functioning of the body or degenerative disease. Veterinarians' assessments of pain were thus linked with these medicalised framings, along with aspects such as their own personal experiences of pain. The context of owners' individual relationships and everyday interactions with their horse is also reported to shape perceptions of pain (findings arising from our ethnographic study are presented elsewhere). As in our study, differing owner and veterinarian views on the presence of pain or significance of suffering in horses is reported.^5^, ^32^ These differing relationships with an animal may account for such varying understandings.

Veterinarians gathered information during the course of the consultation, however, these did not rely heavily upon discussions about the meaning(s) of a horse's behaviour or explicit conversations about pain or its monitoring. Veterinarians prioritised certain issues of concern and were more likely to raise pain as an issue where they perceived a lack of understanding by the owner. This suggests that current approaches can create tensions for early and ongoing discussions about the animal's wellbeing, of which pain may be a concern. Furthermore, while the owner's knowledge of their animal was considered to theoretically be useful with regards to formulating a plan for veterinary care, findings reflect those of Morgan,^33^ who reported that veterinarians made judgements about the reliability and trustworthiness of information provided by owners. However, in human health care it is reported that parents may represent knowledge about their child, including experiences of pain, in different ways to medical practitioners.^34^ It is important for veterinarians to embrace the multitude of contexts, values and concerns that stem from differing horse‐human relationships, and allow for different understandings or explanations related to the animal's wellbeing to be explored in consultations. As Pols discusses,^31^ relational practices shape problems and ways of dealing with them, and they bring with them certain associated values. The ways in which medical practices act to categorise bodies, health and disease,^30^ can generate difficulties in access to health care for patients, for example if their experiences do not appear to fit within these medical constructs.^35^ Thus, acting as a barrier to pain management. We suggest that this study offers insights that may support reflection by individuals and veterinary teams, and bring to the fore taken‐for‐granted attitudes and practices that may be at times creating tensions for pain management. Alongside attempts to work more collaboratively with owners we suggest this can support ‘best interests’ decision making.^36^

Veterinarians presented with animals where they perceive inadequate recognition or management of painful health issues by the owner can provoke moral concern or ethical conflict.^33^, ^36^, ^37^ In our study, broaching issues of pain was acknowledged to be potentially emotive and this may contribute to relational barriers to conversations about its management. Relatedly, Bard et al. discuss the use of euphemisms by veterinarians in place of ‘lameness’ to avoid discomfort in conversations with farmers.^3^ In our study the term was common, however, it was associated with a multitude of meanings and varying inferences about underlying pain. We surmise that the focus on lameness as the ‘matter of concern’ could therefore obfuscate explicit discussions about the subjective experience of the horse. Nevertheless, in some circumstances concerns about significant pain meant veterinarians felt a sense of obligation to elicit a change in owner views or behaviour and they used analogies, explanations or warnings to do so. In a study of veterinarians' communication approaches with farmers, the use of metaphors to simplify understanding of disease processes was observed.^3^ While these types of persuasive approaches are often unsuccessful in motivating change—rather listening and allowing space for self‐reflection are key features of motivational interviewing approaches^38^—it also suggests that conversations about pain may be confined to instances of significant suffering. We suggest that both owners' and veterinarians' knowledge can be valuable in respect of pain management, but navigating conversations may not be straightforward. While the development of formalised assessment tools are ongoing and may assist owners (and veterinarians) in pain monitoring,^39^, ^40^, ^41^ we suggest that the aforementioned factors affect the nature and timing of discussions about pain and are likely to affect the uptake of tools in practice.

While animals cannot request pain relief, veterinarians and owners use a number of strategies for making decisions on their behalf. In this study, veterinarians generally considered that difficulties in pain recognition lay with horse owners. However, identified problems—often acute health matters—often took priority during consultations, and where ‘routine’ consultations were booked in, there was some hesitancy about what an owner may expect from the veterinarian at that time. Doctors' approaches are reported to influence the extent to which discussions regarding patient goal setting pivot around medical constructs.^42^ In our study, whilst veterinarians' concerns were negotiated in light of what they found, issues remained framed within the realms of their personal experience and scientific training. Nevertheless, the (re)presentation of an animal for veterinary involvement is contingent upon what the owner takes away from the interaction, and is related to aspects of the veterinarian‐owner relationship more broadly.^5^ In a context where early management failure can result in difficulty or inability to achieve control over pain,^43^ it is important that not only are owners' concerns managed, but issues of the horse discussed in a timely manner to enable their early and ongoing management. This may also necessitate further education of veterinarians to reinforce ideas such as the importance of prompt treatment of pain, including flare‐ups of chronic issues, and the range of pain management options available.^44^

This study has some limitations. Although data collection took place across different sites and different seasons, there was still a limited time for data collection. The sample included practices that were willing to participate. The predominant reason for recruitment failure was lack of response to the study invitation, and one practice declined to participate following discussion with RS about what the study would involve. The selection of consultations to attend was based on the desire to include a range of veterinarians and consultation types and driven by theoretical sampling. This affected the nature of data collected and the owners subsequently recruited. Some owners did not take part in the interview stage due to a lack of response (following phone call, text or email) and also due to the selection of cases by RS based on theoretical sampling approaches and practicalities of doing interviews alongside other data collection. Ethnographic studies are often lengthier than the periods of observation in this study. Studying one practice for a more prolonged period of time may have produced an understanding of veterinarians' everyday lives as well as how their interactions with horse owners shaped relationships over time. However, the intermittent nature of veterinarian‐owner interactions meant that in order to understand this intersection of the lives of owners and veterinarians, the collection of data in this way was appropriate.

We suggest that much can be gained through the adoption of ethnographic approaches in future veterinary research. Increased attention to animal subjectivity and agency could be further incorporated, for example, through the use of multispecies ethnographic methods.^45^ These approaches open up the possibility to explore and shed new light on wide‐ranging issues affecting animals and people across the profession.

In conclusion, this study contributes empirical findings with implications for veterinary practice. It demonstrates the ways through which animal bodies and subjective experiences are shaped and given meaning through interactions, and the implications for decisions regarding an animal's interests. In highlighting the drivers of decision making in pain diagnosis and treatment, we have outlined opportunities for reflection. Greater attention to the diverse contexts in which animals experiencing pain may be presented to a veterinarian, as well as reflection on pain assessment practices and a focus on veterinary education, may all lead to improved care and horse welfare. The significant impact of current practices on the success of chronic pain management suggests that prompt attention is required.

## FUNDING INFORMATION

This study was supported by The Horse Trust, grant number G2026.

## CONFLICT OF INTEREST STATEMENT

The authors declare no conflicts of interest.

## AUTHOR CONTRIBUTIONS

**Rebecca Smith:** Conceptualization; methodology; software; data curation; investigation; writing – original draft; writing – review and editing; validation; formal analysis; project administration; resources; funding acquisition. **Elizabeth Perkins:** Conceptualization; funding acquisition; methodology; writing – review and editing. **Gina Pinchbeck:** Conceptualization; funding acquisition; writing – review and editing. **Joanne Ireland:** Conceptualization; funding acquisition; writing – review and editing; project administration.

## DATA INTEGRITY STATEMENT

Rebecca Smith had full access to all the data in the study and takes responsibility for the integrity of the data and the accuracy of the data analysis.

## ETHICAL ANIMAL RESEARCH

The study was reviewed and approved by the University of Liverpool Veterinary Research Ethics Committee.

## INFORMED CONSENT

Informed consent was obtained.

## Supporting information


**Table S1.** Veterinary practice details.

**Table S2.** Owner interview participant details.

**Text S1.** Interview topic guides.

## Data Availability

Open data sharing is not applicable to this article due to the qualitative nature of the data in this study.
