# Supplementary material for: Drivers of decision making in pain diagnosis and treatment: Findings from an ethnographic study of veterinary practice
Source: Equine Vet J. 2025 Jul 27;58(3):824–36. doi: 10.1111/evj.14562 (PMC13041593; doi:10.1111/evj.14562)
Supplement: Supplementary file 1 — Table S1. Veterinary practice details. [file EVJ-58-824-s001.pdf]

Equine Veterinary Journal BEVA

Table S1: Veterinary practice details.

**Practice site identifier**	**Practice details**	**Approximate hours of observation**	**Consultations observed**	**Informal/ethnographic veterinarian interviews**	**Recorded semi-structured veterinarian interviews**	**Recorded semi-structured owner interviews**
A	RCVS accredited Equine General Practice (University)	27	9	2 (1 female, 1 male)<br><br>Employed veterinarians, between 8 and over 25 years graduated.	2 (1 female, 1 male)	4
B	Corporate-owned, RCVS accredited Equine Veterinary Hospital	53	14	11 (8 female, 3 male)<br><br>Partners in the practice and employed veterinarians. Range of experience from 1 year (interns) to over 35 years graduated.	3 (2 female, 1 male)	3
C	Independently owned mixed practice. RCVS accredited Equine Veterinary Hospital	50	14	10 (6 female, 4 male)<br><br>Partners in the practice and employed veterinarians. Some treated horses only, some treated horses as well as companion and production animals. Range of experience from 1 year (interns) to over 30 years graduated.	6 (3 female, 3 male)	8 (1 written)
D	Independently owned, RCVS accredited Equine General Practice	66	10	7 (6 female, 1 male)<br><br>Partners in the practice and employed veterinarians. Range of experience from 2 to 23 years graduated.	7 (6 female, 1 male)	10
