# Supplementary material for: Drivers of decision making in pain diagnosis and treatment: Findings from an ethnographic study of veterinary practice
Source: Equine Vet J. 2025 Jul 27;58(3):824–36. doi: 10.1111/evj.14562 (PMC13041593; doi:10.1111/evj.14562)
Supplement: Supplementary file 2 — Table S2. Owner interview participant details. [file EVJ-58-824-s002.pdf]

Table S2: Owner interview participant details.

Practice	Consultation type (as presented)	Horse housing premises	Horse details
A	Follow up consultation for steroid injection	DIY livery	28-year-old Cob gelding. Previously used by owner for driving and mixed leisure pursuits, stopped riding last 5 months
A	Sedate for farrier, check sarcoid and additional pony presented during visit for pain and hoof abnormality	DIY livery	9 horses in total. Hacking and carriage driving
A	Lameness and throwing head up	DIY livery	14-year-old mare. Also owns a 21-year-old mare as well as donkeys
A	Vaccination	DIY livery	17-year-old gelding, 10-year-old gelding and previously owned a 30-year-old gelding
B	Admit horse to the hospital for stifle injection	Kept on a farm near house	20-year-old Thoroughbred cross, gelding. Hunter, being brought back into work over the autumn
B	Lameness	Private home premises	7-year-old and 12-year-old geldings. Both used for eventing
B	Review intermittent lameness	DIY livery	7-year-old Irish Sports Horse, gelding. Leisure/pleasure
C	Put to sleep (euthanasia)	DIY field and stables for 3 horses	Cob, mare. Companion horse. Also owned a 7-year-old retired gelding and 2 horses kept at another premises
C	Dental and blood tests (Adrenocorticotropic hormone/ACTH)	Rented stables and fields at farm near to home	25-year-old Connemara gelding
C	Blood tests (ACTH)	DIY livery	21-year-old Thoroughbred cross, gelding. Leisure/pleasure. Also own another horse
C	Lameness	DIY livery	24-year-old Welsh Cob mare. Leisure/pleasure
C	Vaccination and two prescription check-ups	Large DIY livery	21-year-old Standardbred gelding, retired, 26-year-old mare and 13-year-old mare
C	Presented at hospital for radiographs for laminitis review	Kept at own home	10-year-old Connemara cross, mare. Pony club competition. Housed with companion pony
C	Presented at hospital, booked into diary as ‘MOT’ [check-up]	Farm livery	9-year-old Thoroughbred cross, gelding. Eventing
C	Behaviour changes when ridden	Large DIY livery	8-year-old sports horse, mare. Leisure/pleasure (Written interview responses)

C	Presented at hospital. Re-examine post kissing spine surgery	Large DIY livery	10-year-old warmblood, gelding. Dressage, showjumping, hacking. Previously owned an 18-year-old Irish Sports Horse, gelding
D	Re-scan ligament	DIY livery	13-year-old Welsh Cob, mare. Leisure, hacking
D	Ridden assessment/performance review for potential lameness	DIY livery<br>(2 owners and livery yard owner interviewed)	6-year-old mare, backed last year. Hacking, school work. Also own another retired gelding
D	Prescription check-up and sedate for dental	Private rented stables and field	28-year-old Irish Sports Horse, gelding and 11-year-old Thoroughbred cross gelding. Leisure, hacking
D	Re-scan following tendon injury	DIY livery	10-year-old Thoroughbred, gelding. Leisure, hacking
D	Radiographs following emergency veterinary visit	Kept at own home	34-year-old Arab, gelding. Retired
D	Lameness investigation (clinic)	Home premises	10-year-old gelding. Eventing
D	Medicate hock and back	Assisted livery	17-year-old Irish Draught cross warmblood, gelding. Hacking
D	Re-examine for stifle injection, second lame horse, vaccinate 7 horses	Riding centre for non-able-bodied riders	Multiple horses owned, generally reported to be older animals
