# Supplementary material for: Drivers of decision making in pain diagnosis and treatment: Findings from an ethnographic study of veterinary practice
Source: Equine Vet J. 2025 Jul 27;58(3):824–36. doi: 10.1111/evj.14562 (PMC13041593; doi:10.1111/evj.14562)
Supplement: Supplementary file 3 — Text S1. Interview topic guides. [file EVJ-58-824-s003.pdf]

Equine Veterinary Journal BEVA

Text S1: Interview topic guides.

Veterinary surgeon

Experiences

- Could you tell me about your background working as a vet?
- What is your caseload like? (Prompts: What is the setup of the practice? Are clients able to contact you directly or via a reception team? Do you see your clients regularly for routine and emergency visits? How do you manage on-call?)

Pain

- How would you know if a horse had orthopaedic-related pain? (Prompts: What do you rely on to assess a horse's pain? Do you use any particular scales/frameworks? Would this differ in certain cases? What is the owner's role?)
- Are there any cases we've seen since I've been with you where you feel that a horse has had this type of pain? (Prompts: How did you feel about the outcome of that visit? How do you feel about the conversations that took place between you and the owner? Were you able to discuss your concerns about the horse with the owner? Did you have any concerns about what was discussed e.g. possible diagnosis/investigations/treatment plans?)
- What does chronic pain mean to you? (Prompts: How would you know if a horse had chronic pain?)
- What are the most common orthopaedic-related cases that you see? (Prompts: Are these chronic cases? What are your main concerns in these types of cases? Are these the same concerns as owners have? Do you have any difficulties in assessing a horse's pain? How do you approach conversations about chronic pain?)
- How would you go about managing chronic pain? (Prompts: What have you learnt is successful or unsuccessful? What do you feel about the treatment options you have available to you? What would prompt you to approach these cases in a particular way? Would any factors limit your ability to manage a horse successfully?)

- How would you go about monitoring a horse's level of pain? (Prompts: Do you think that your approaches differ in any way from an owner? How might monitoring be improved? How are clinical records used to do this?)
- Are there any instances where you think that chronic pain is necessary or acceptable? (Prompts: What are these contexts? How might you go about resolving such issues?)

Quality of life and euthanasia

- What do you think about chronic pain in relation to quality of life? (Prompts: Has your understanding of chronic pain has changed over time? What has changed your views? Does this make you act any differently? Do you have any regrets about how you have managed previous cases?)
- How are these beliefs factored into your clinical decision-making? (Prompts: Are there any differences in how you and your colleagues make quality of life judgements? Has this caused any conflict?)
- How are these beliefs factored into advising owners about euthanasia?

Closing questions

- Is there anything that you might not have thought about before that has occurred to you during this interview?
- Is there anything else you would like to add?
- Is there anything you would like to ask me?

<u>Horse owners</u>

Scene setting/experience

- Can you tell me about you and your horse?
- Could you tell me about your reason for booking the vet visit that I attended? (Prompts: Follow-up on specific points if mentioned e.g. signs of first noticing a problem, other

strategies/advice sought in attempts to remedy the problem. If it was a routine visit, ask what they had hoped to get from the visit)

Reference to observed consultation

- How do you feel about the outcome of the consultation? (Prompts: Were you able to discuss any concerns you had about your horse with the vet? How do you feel about the conversations that took place between yourself and the vet? Did you have any concerns about what was suggested e.g. possible diagnosis/investigations/treatment plans?)
- What is your plan for managing the problem going forward?
- How do you see the role of the vet in your horse's care? (Prompts: What kind of relationship do you have with the vet? Have you seen this particular vet previously?)

Management & care

- Could you describe any accommodations you have made for your horse? (Prompts: What made you decide to change your management practice? How do you feel that has gone?)
- Have you ever had a concern about your horse's behaviour? If so, how did you try to resolve it? (Prompts: Have you changed your approaches over time? Where did/would you go to for advice?)

Pain

- How would you know if your horse was in pain? (Prompts: What do you think is important to this process? Do you think particular people are better at this than others? Do you have any difficulties in deciphering if your horse is in pain?)
- How do you know where the source of the pain is? (Prompts: How would you go about finding out?)
- If you thought your horse was in pain, what would you do? (Prompts: If use of NSAIDs is mentioned, how are these obtained? If employing a physiotherapist or chiropracter etc., how did you decide who to employ?)
- What does 'long-term' pain mean to you? Is this different in any way from 'chronic' pain?

- Have you ever thought that someone else’s horse was in long-term pain? (Prompts: What do you think this means for the horse? Are there any occasions where long-term pain might be acceptable for a horse?)
- If you though your horse was in long-term pain would this make you think any differently about how you would manage them? (Prompts: Would it make you think differently about their quality of life? What about in relation to end-of-life decision-making?)

Closing questions

- Is there anything that you might not have thought about before that has occurred to you during this interview?
- Is there anything else you would like to add?
- Is there anything you would like to ask me?
